# Zinc Sulphate Test for Uric Acid

**Published:** 1913-11

**Authors:** 


Zinc Sulphate Test For Uric Acid. Ganassimi, quoted in *Critic and Guide*, states that if an aqueous solution is added to uric acid or an alkaline urate, white, basic zinc urate is precipitated. On exposure to air it gradually becomes greenish or bluish. Albumins do not interfere with the reaction. He thinks this reaction will supplant the well known murexid test. It can be applied to blood.

